# Establishing primary surface rupture evidence and magnitude of the 1697 CE Sadiya earthquake at the Eastern Himalayan Frontal thrust, India

**DOI:** 10.1038/s41598-020-79571-w

**Published:** 2021-01-13

**Authors:** Arjun Pandey, R. Jayangondaperumal, György Hetényi, Rao Singh Priyanka, Ishwar Singh, Pradeep Srivastava, Hari B. Srivastava

**Affiliations:** 1 Wadia Institute of Himalayan Geology, Dehradun, India; 2 Department of Geology, School of Earth Sciences, Central University of Tamil Nadu, Thiruvarur, 610005 India; 3 Institute of Earth Sciences, University of Lausanne, Lausanne, Switzerland; 4 Department of Geology, Banaras Hindu University, Varanasi, India

**Keywords:** Geodynamics, Geology, Seismology, Tectonics

## Abstract

Historical archives refer to often recurring earthquakes along the Eastern Himalaya for which geological evidence is lacking, raising the question of whether these events ruptured the surface or remained blind, and how do they contribute to the seismic budget of the region, which is home to millions of inhabitants. We report a first mega trench excavation at Himebasti village, Arunachal Pradesh, India, and analyze it with modern geological techniques. The study includes twenty-one radiocarbon dates to limit the timing of displacement after 1445 CE, suggesting that the area was devastated in the 1697 CE event, known as Sadiya Earthquake, with a dip-slip displacement of 15.3 ± 4.6 m. Intensity prediction equations and scaling laws for earthquake rupture size allow us to constraints a magnitude of Mw 7.7–8.1 and a minimum rupture length of ~ 100 km for the 1697 CE earthquake.

## Introduction

**Figure 1:**
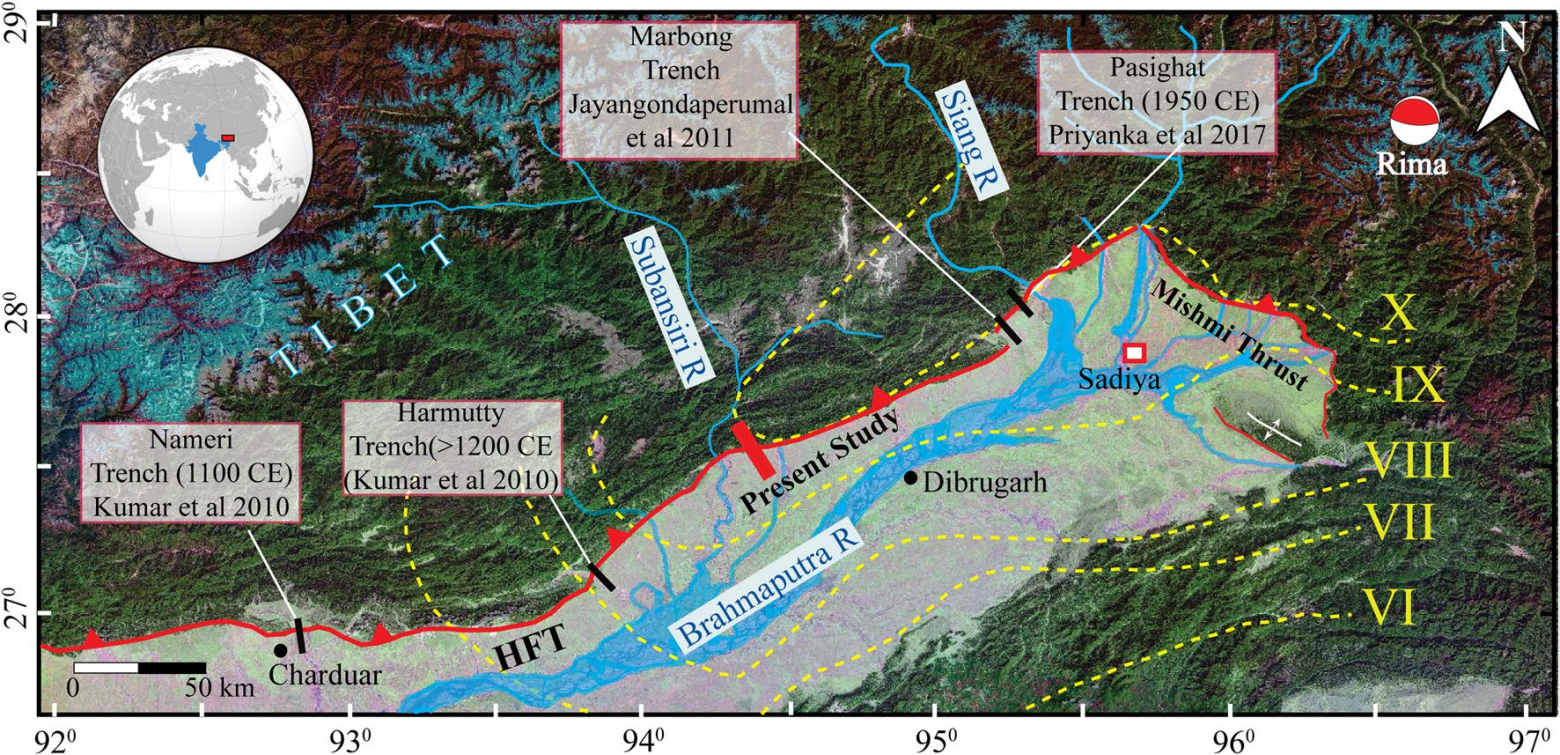
Regional map of the eastern Himalaya draped on Shuttle Radar Topographic Mission (SRTM) and Landsat satellite imagery. Solid black lines show locations of previous trenches. The solid red line shows the trench location of this study. Yellow dotted lines with Roman numerals are the 1950 CE earthquake isoseismals^51^. Red beach ball shows location of 1950 CE earthquake epicenter^32^. Red square with white fill is the reported maximum actual damage during the 1697 CE Sadiya earthquake^1^. The moment tensor solution (red beach ball) of the 1950 CE earthquake by Coudurier-Curveur et al.^18^ shows a double couple solution for HFT and Mishmi Thrust. Map was prepared in Arc Gis v10.3 using SRTM GTOPO 30 m and Landsat satellite imagery available at http://glcfapp.glcf.umd.edu/data/srtm/description.shtml. Artwork was done in Adobe Illustrator CS5.

Historical archives document massive destruction in the eastern Himalaya during closely time-spaced earthquakes in Late-Medieval times^1,2^. These earthquakes sometimes ruptured the Himalayan Frontal Thrust (HFT) at the surface, or remained blind, like the 2015 Gorkha earthquake^3^. However, blind events rupture the down-dip segment of the MHT (Main Himalayan Thrust) and thus transfer stress to the locked up-dip portion of this megathrust, which eventually breaks in subsequent, great earthquakes^4^. Despite an increasing number of paleoseismological studies in the central Himalaya during the last decade^5–12^, only a few investigations have been conducted along the eastern Himalayan front, and the studied trench sites are rather sparsely spaced at ~ 50 to ~ 200 km apart^13–17^. The area includes Bhutan–Arunachal Pradesh segment of the Himalaya, which lies in the mesoseismal zone of 1950 CE great earthquake^17,18^ (Fig. 1). Large spacing amongst the sites causes uncertainties associated with locations, surface rupture extents, chronologies, and magnitudes of historical earthquakes. Therefore, assessing seismic hazards associated with earthquakes on the HFT and determining its seismic behaviour in the eastern Himalaya has proven difficult. To better understand the seismogenic potential of the east Himalayan front, we conducted a palaeoseismological investigation between the Subansiri and Siang river valleys at Himebasti village in Arunachal Pradesh, India.

### Trench site at Himebasti

**Figure 2:**
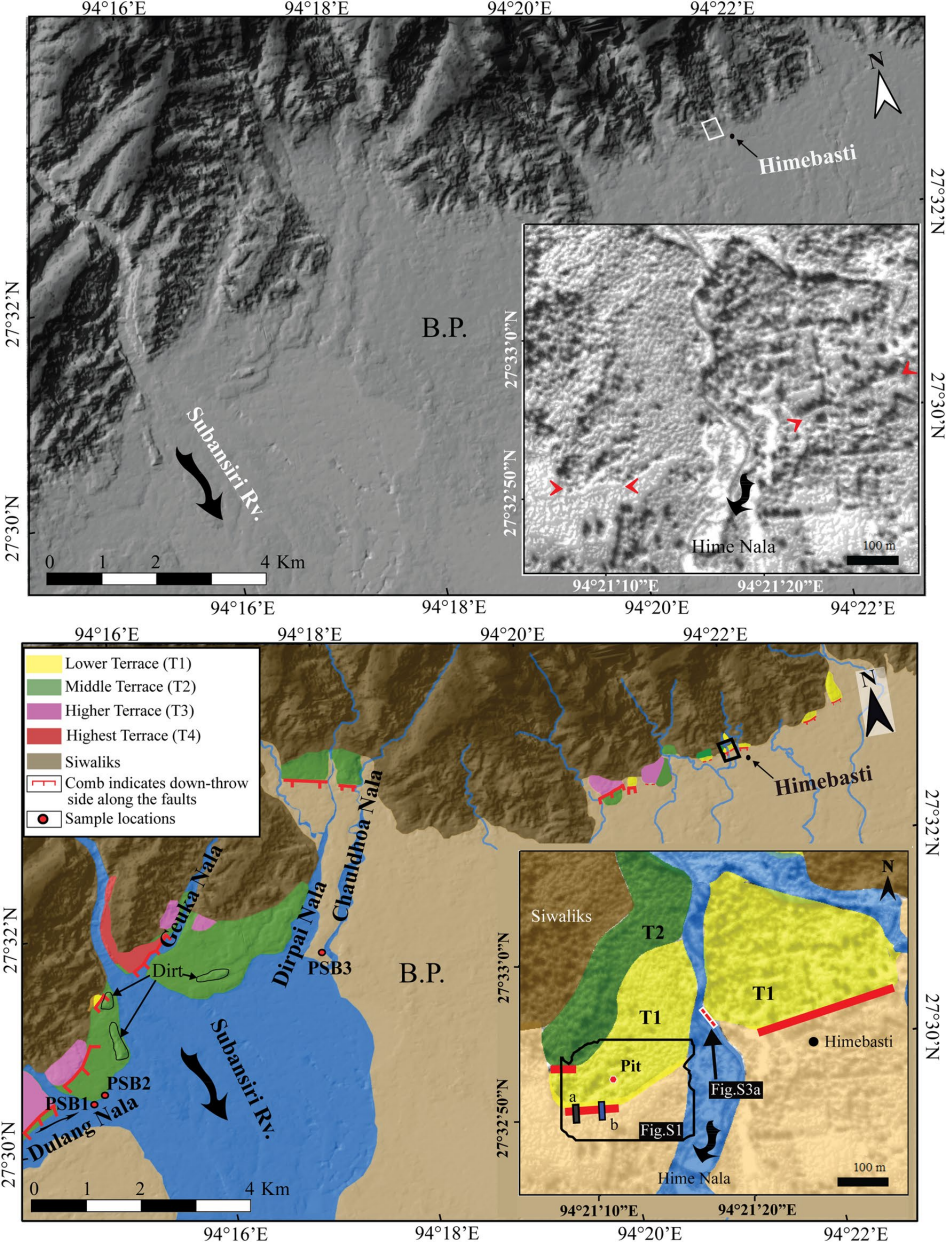
Cartosat-1A digital elevation model showing the regional tectonic-geomorphic map of the trench site at Himebasti. (Top) Un-interpreted Cartosat-1A digital elevation model showing the extent of different terraces. (Inset) the small red arrows in the inset are pointing at the strike of the scarp. (Bottom) mapped terraces are differentiated by different colours in and around the study area. (Inset) Detailed tectonic-geomorphic map of the trenched site at Himebasti village. A small black outlined polygon box depicts the location of Figure S1. Letters 'a' and 'b' denotes the locations of the previous^20^ and present trench sites, respectively. BP—Brahmaputra Plain. Maps were prepared using Cartosat-1A imageries purchased from http://www.nrsc.gov.in (Source: NRSC, ISRO/DOS) and processed in SOCET GXP version 4.1.0 software and artwork in Adobe Illustrator CS5 software.

Himebasti is a small village in Arunachal Pradesh, India, located along the foothills of the eastern Himalayan mountain front, ~ 7 km east of the Subansiri River (Figs. 1, 2). Here several active faults truncate fluvial terraces along the HFT at the exit of the Subansiri River and its tributaries (Fig. 2). The mountain front comprises Siwalik rocks of the Dafla Formation, which includes massive sandstone with a minor proportion of conglomerates^19^. Numerous dried and active drainages were observed near the study area, running orthogonal to the mountain's structural grain.

The study area was mapped using the Cartosat-1A digital elevation model purchased from http://www.nrsc.gov.in (Source: NRSC, ISRO/DOS) with a resolution of 5 m (Fig. 2). Four flights of relict terraces are preserved in the area and characterized based on their respective elevation from the current river grade. They are, in order of decreasing elevation, highest (T4), higher (T3), middle (T2), and lower terraces, respectively (T1) (Fig. 2). The T4 terrace is a strath terrace that sits at the height of ~ 39 m from the current Subansiri River grade and is cut by an active fault. The T3 terrace is preserved on either bank of Geuka Nala and the eastern bank of the Dulang Nala, and it sits at the height of ~ 27 m from the current Subansiri River grade (Fig. 2). The T2 terrace is mapped at the exit of the Subansiri River. At the trench site, the T2 terrace is affected by a fault scarp and sits at the height of ~ 20 m from the current grade of a small local stream, Hime Nala (Fig. 2). The T1 terrace is preserved near the trench site and has a height of ~ 12 m from Hime Nala (Fig. 2). Terrace T1 is uplifted by an active fault that has generated a large fault scarp, with a vertical offset of ~ 6.8 m and a lateral span of ~ 0.5 km, bounded by ephemeral streams on either side (Fig. 2). Field observations near the trench site show abnormal footwall incision just south of the fault scarp. A Siwalik strath of ~ 1.2 m is seen at the eastern bank of Hime Nala (Supplementary Fig. S3). We observed many dried and beheaded channels near the trench site.

**Figure 3:**
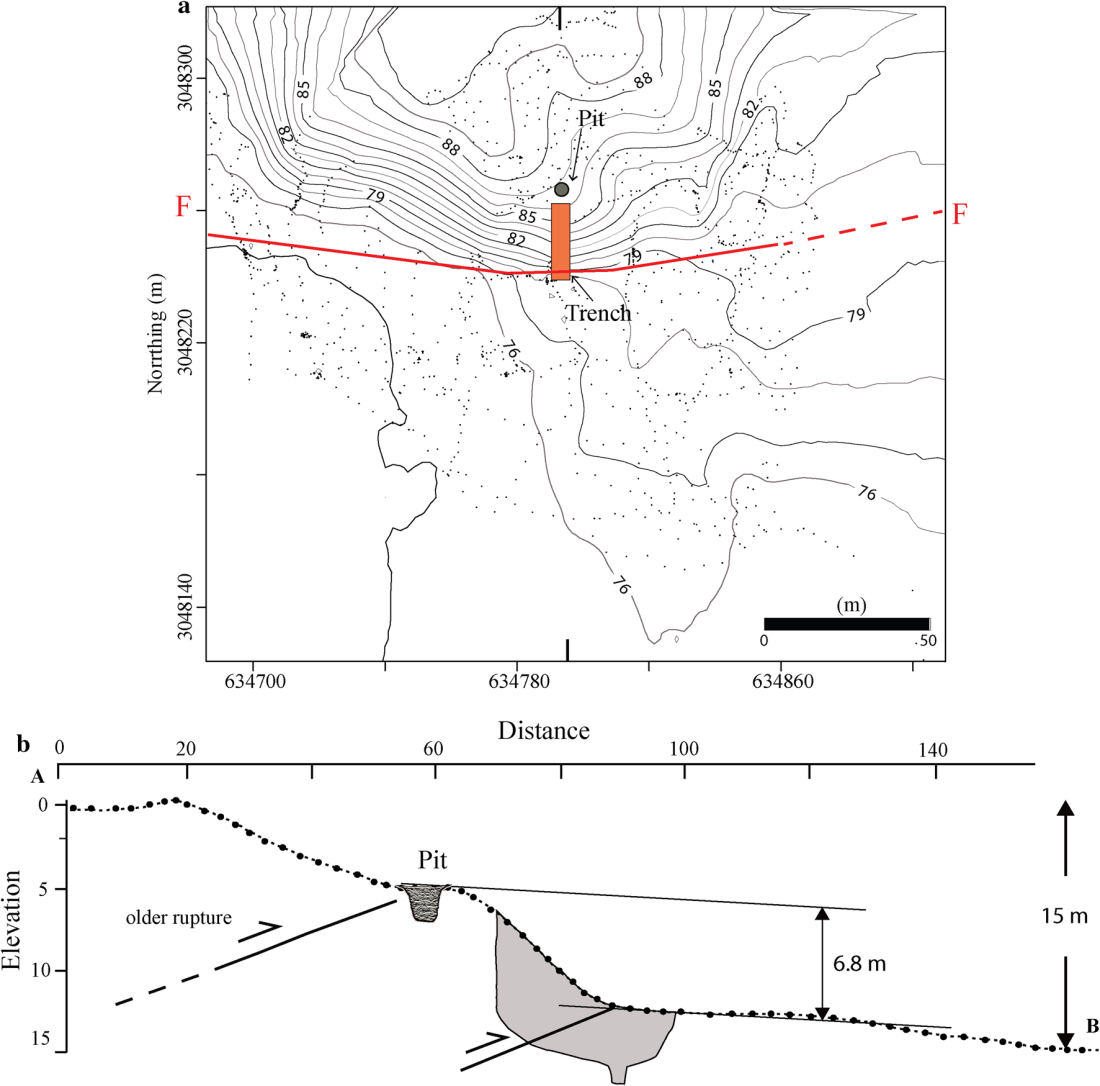
Topographic map of Himebasti trench site, contours on the map are at 1 m interval based on a survey using Real-Time Kinematic Global Positioning System (RTK-GPS) in UTM 46N coordinates. An orange rectangle denotes the location of a N-S oriented trench (Top). RTK-GPS N-S (along AB) topographic profile across the fault scarp and projection of the Himebasti trench is shown in the bottom figure. The excavated pit has been projected over the ~ 6.8 m high fault scarp. Location of the pit is shown in Fig. 2, and field photograph is shown in Supplementary Fig. S3. Contour map and profile were prepared in Leica Geo Office v7.0 programme and artwork in Adobe Illustrator CS5 software.

The fault scarp was mapped in the field using a Real-Time Kinematic Global Positioning System (RTK-GPS), and an aerial view was captured using an Unmanned Aerial Vehicle (UAV) to orient the paleoseismic trench orthogonal to the strike of the scarp (Fig. 3 and Supplementary Fig. S1). A micro-topographic map of the trench site was generated, in conjunction with a scarp profile (Fig. 3) that shows two scarps with vertical separations of ~ 8.2 m and ~ 6.8 m, respectively. A paleoseismic trench was placed a few meters east of a previous trench^20^ across the ~ 6.8 m high, steeply sloping (36°), south-facing fault scarp (Fig. 3 and Supplementary Fig. S2). A pit was excavated on the scarp's hanging wall for correlation of both hanging and footwall units observed in the trench exposures (Supplementary Fig. S3).

## Structural and stratigraphic relationships in the trench exposures

### Trench exposure

**Figure 4:**
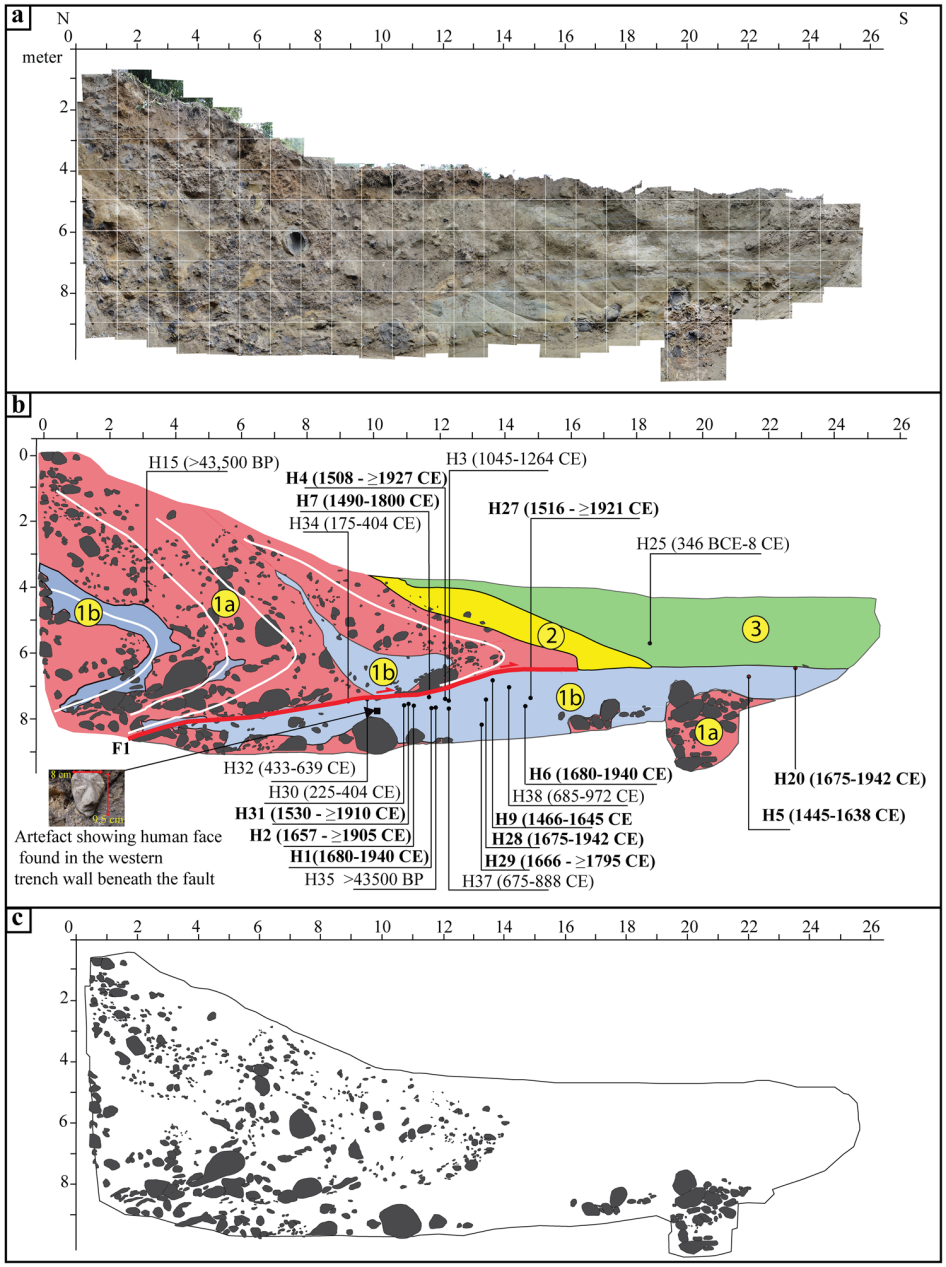
(**a**) Photomosaic of the excavated trench exposures at Himebasti site showing 1 × 1 m grids. Horizontal and vertical scales are in meters. (**b**) Illustrative log of the trench exposures shown in (**a**) showing different stratigraphic units (numbers in yellow circles) concerning the faulting events (red) and the sample locations showing 2-sigma values. A solid black square depicts the location of an artifact of human face. (**c**) Illustrative log of the trench exposure for a better understanding of the fabric. Mosaic of the trench photographs was done in Adobe Photoshop CS5 software and artwork in Adobe Illustrator CS5 software.

Excavation of a paleoseismic trench (~ 26 m long, 9 m deep, and 4 m wide) at Himebasti exposed fluvial deposits deformed by ‘F1’, thrust along an ~ NE dipping fault zone (Red line in Fig. 4b). The associated photomosaic (Fig. 4a) and digitized log (Fig. 4b) depict three stratigraphic units (units-1, 2, and 3) observed in the trench exposures, which were differentiated based on grain size and depositional facies variations. A remarkable folding fabric is visible in the hanging wall gravels, which is visible in Fig. 4c. Unit 1 consists of two stratigraphic units, which are referred to as Unit-1a and 1b. Unit-1a is the oldest, faulted unit comprising rounded, sub-rounded and sometimes angular to sub-angular dark grey to brown Siwalik sandstone. The clast size varies from 2 to 15 cm (Fig. 4a). Unit-1a was observed in the footwall underlying unit-1b in a deeper pit excavated between the 19 m and 21 m horizontal marks of the trench exposures (Fig. 4b). Unit-1b comprises massive grey silty clay. This unit is intermixed with unit-1a between the 8 and 9 m horizontal marks. Atop, unit-1b is deformed and appearing as thin grey silty sand with numerous liquefied features. Beneath this, the unit appears as massive silty clay with numerous burned rootlets. Unit-2 consists of variegated dark brown and light-yellow silty clay with brownish-yellow sand with few occasional pebbles. Unit-3 comprises of cross-bedded brownish oxidized sand which is seen in the clean white sand.

Units 1a and 1b in the hanging wall are truncated by a shallow north east-dipping (∼ 9°NE–11°NE) basal thrust fault marked F1 in Fig. 4b. Unit 1b is an overbank deposit. A sandstone artefact carved with a human face was recovered beneath the fault zone F1 from unit-1b of the western trench wall exposures, suggests that this unit is a 'paleosurface or paleosol-1’ (Fig. 4b). The unit-1 (1a and 1b) is folded, overturned, and thickened in the hanging wall forming an asymmetrical, south-verging fold (Fig. 4 and Supplementary Fig. S4). Thickening and overturning of units 1a and 1b in the hanging wall are the result of folding due to drag along the F1 fault strand during the coseismic displacement. Part of unit-1b is found intercalated within unit-1a as liquefied patches between the 2 m and 5 m horizontal marks and is folded in association with the fault strand F1 (Fig. 4, Supplementary Figs. S4, and S5). The chaotic texture of unit-1b, between the 8 m and 13 m horizontal marks, replicate the warping and bulldozing of material along the fault F1. Unit-2 is colluvial wash derived from faulted units (i.e., 1a and 1b) and is formed by unroofing of the material from the hanging wall of the scarp. Unit-3 is deposited because of the aggradation by local streams after the formation of the fault scarp. Unit-2, along with unit-3, post-dates the most recent displacement. We excavated a 2 m deep pit on the hanging wall above the trench that exposed both units 1a and 1b (Fig. 3 and Supplementary Fig. S3) and they are seen in the footwall suggesting a coseismic vertical offset after the deposition of unit-1.

### Faulting event at Himebasti

**Figure 5:**
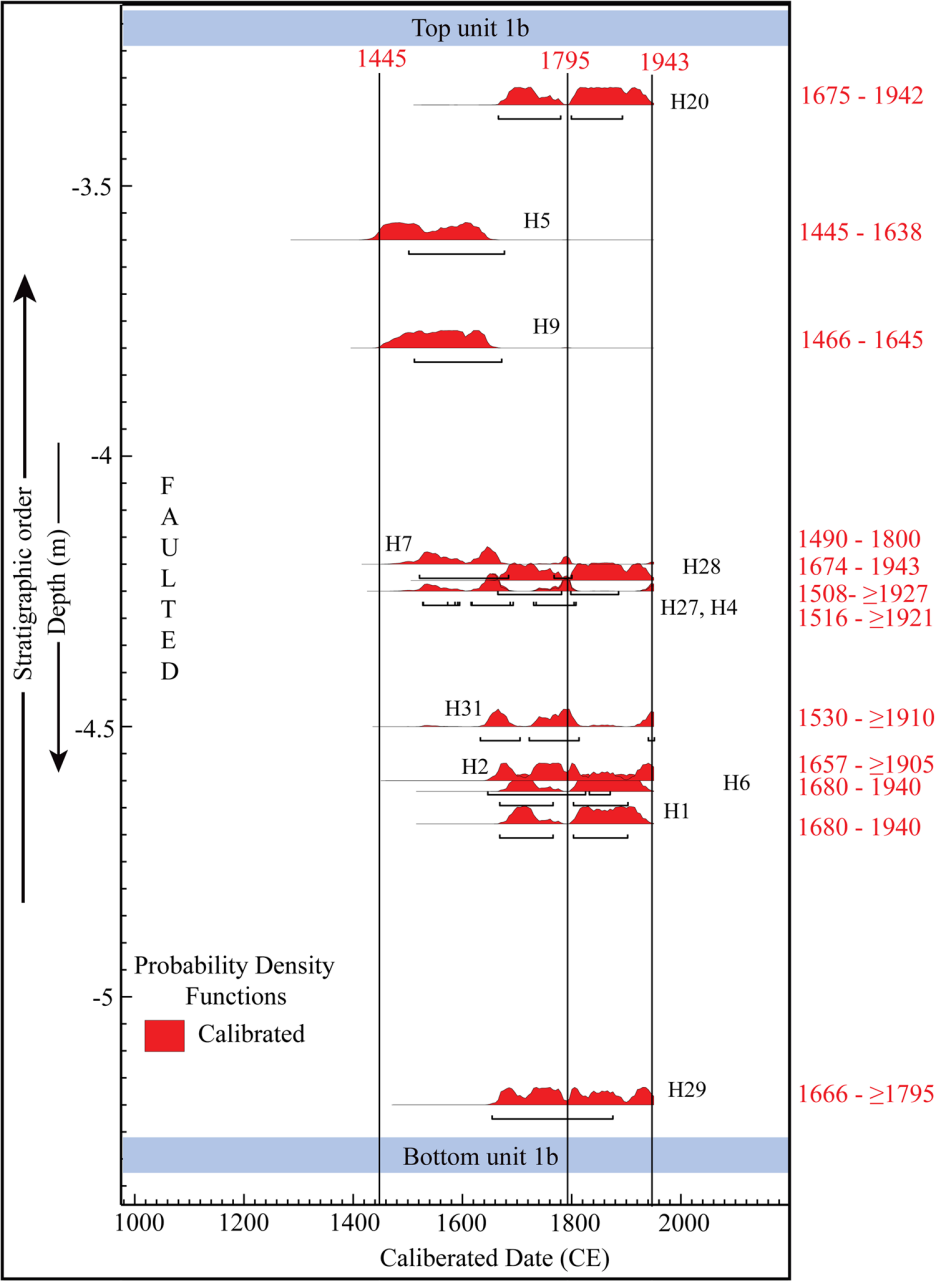
Probability density function (PDF) plot of radiocarbon samples obtained from the Himebasti trench exposure plotted in an age-depth model showing span of calendar ages. Plot generated in Ox-Cal v4.4.2 (https://14C.arch.ox.ac.uk/oxcal/OxCal.html)^21^ with the IntCal13 atmospheric curve of Reimer et al.^52^. Artwork was performed in the Adobe Illustrator CS5.

Twenty-one charcoal samples obtained from the trench exposures were dated using Accelerator Mass spectrometry (AMS) dating to constrain the timing of faulting events in the region. Detrital charcoal samples collected from the different units have been calibrated and plotted in stratigraphic order in an age-depth model using the Ox-Cal online programme (Ox-Cal v4.4.2 (https://14C.arch.ox.ac.uk/oxcal/OxCal.html)^21^ (Fig. 5, Table 1). Table 1 shows the 2-sigma values of all the detrital charcoal samples collected from the trench exposure. Ox-Cal calibrates the radiocarbon ages based on probability density functions and gives us a probable range of calendar ages for a specific sample (Fig. 5), providing 2-sigma (95.4%) confidence index of the calendar ages (Table 1, Fig. 5). However, this 95.4% probability is further break up into smaller subsets of ages with different relative probability percentages (Table 1). Table 1 shows the breakup of ages for each sample.

**Table 1:** AMS Radiocarbon (^14^C) dates from detrital charcoals collected from the Himebasti trench.

Unit^a^	Sample ID	Lab Code^b^	Uncalibrated conventional radiocarbon age^c^	∂3C repo rted^d^	Calibrated ages (calendric, 2σ, 95.4%)^e^	Relative probability distribution percentage (%)
**Eastern wall, Himebasti Trench**						
1a	H15	Beta-279175	> 43,500 BP	− 25.6	> 43,500 BP	–
1b	H35	Beta-279177	> 43,500 BP	− 24.8	> 43,500 BP	–
1b	H30	Beta-279168	1760 ± 40 BP	− 26.9	CE 225–404	95.4
1b	H32	Beta-279170	1520 ± 40 BP	− 25.1	CE 433–610 CE 619–639	90.6 4.8
1b	H37	Beta-279167	1230 ± 40 BP	− 10.7	CE 675–888	95.4
1b	H3	Beta-279176	870 ± 40 BP	− 25.0	CE 1045–1086 CE 1093–1105 CE 1120–1264	15.4 2.0 78
**1b**	**H5**	**Beta-281522**	**370** ± **50 BP**	**NA**	**CE 1445–1638**	**95.4**
**1b**	**H9**	**Beta-279181**	**330** ± **40 BP**	− **25.7**	**CE 1466–1645**	**95.4**
**1b**	**H7**	**Beta-281500**	**270** ± **40 BP**	− **29.0**	**CE 1490–1676** **CE 1743–1750** **CE 1765–1800**	**85.4** **0.7** **9.4**
**1b**	**H4**	**Beta-281502**	**250** ± **40 BP**	− **24.9**	**CE 1508–1594** **CE 1618–1686** **CE 1732–1806** **CE ≥ 1927**	**24.4** **41.4** **26.1** **3.5**
**1b**	**H27**	**Beta-281503**	**240** ± **40 BP**	− **30.3**	**CE 1516–1590** **CE 1620–1690** **CE 1728–1809** **CE ≥ 1921**	**15.0** **40.1** **34.4** **5.9**
**1b**	**H31**	**Beta-281501**	**210** ± **40 BP**	**NA**	**CE 1530–1539** **CE 1635–1699** **CE 1722–1814** **CE 1835–1885** **CE ≥ 1910**	**0.6** **28.8** **47.8** **4.8** **13.3**
**1b**	**H2**	**Beta-281523**	**170** ± **40 BP**	**28.0**	**CE 1657–1711** **CE 1718–1822** **CE 1831–1894** **CE ≥ 1905**	**18.4** **42.6** **16.4** **18.1**
**1b**	**H29**	**Beta-281504**	**150** ± **40 BP**	− **30.2**	**CE 1666–1783** **CE ≥ 1795**	**41.9** **53.6**
**1b**	**H28**	**Beta-281518**	**120** ± **40 BP**	− **31.7**	**CE 1674–1766** **CE 1774–1776** **CE 1799–1943**	**31.7** **0.5** **63.3**
**1b**	**H20**	**Beta-279169**	**110** ± **40 BP**	− **28.8**	**CE 1675–1744** **CE 1750–1765** **CE 1799–1942**	**26.9** **3.1** **65.4**
**1b**	**H6**	**Beta-279179**	**90** ± **40 BP**	− **27.9**	**CE 1680–1740** **CE 1753–1763** **CE 1800–1940**	**26.3** **1.4** **67.7**
**1b**	**H1**	**Beta-279180**	**90** ± **40 BP**	− **29.6**	**CE 1680–1740** **CE 1753–1763** **CE 1800–1940**	**26.3** **1.4** **67.7**
3	H25	Beta-281521	2100 ± 40 BP	28.2	BCE 346–317 BCE 204–32 BCE 17–8 CE	4.7 87.3 3.5
**Drift Wood Samples, Himebasti**						
PSB-1		Beta-279194	170 ± 40 BP	25.1	CE 1657–1711 CE 1718–1822 CE 1831–1894 CE ≥ 1905	18.4 42.6 16.4 18.1
PSB-2		Beta-279195	120 ± 40 BP	26.9	CE 1674–1766 CE 1774–1776 CE 1799–1943	31.7 0.5 63.3
PSB-3		Beta-279196	120 ± 40 BP	25.2	CE 1674–1766 CE 1774–1776 CE 1799–1943	31.7 0.5 63.3

PSB1, 2 and 3, are buried wood logs. All other samples are detrital charcoals.

^a^Refer to respective trench logs for stratigraphic unit designations.

^b^Samples have been analyzed at Beta Analytic (Florida) by accelerator mass spectrometry (AMS). Each number corresponds to the laboratory code for each sample.

^c^Conventional Radiocarbon years BP relative to 1950 CE with 2σ confidence level comprising of counting statistics, reference standard, blank, and random machine error.

^d^The δ13C values are the assumed values when given without decimal places. Values measured for the material itself are given with a single decimal place.

^e^Pre-bomb calendric dates were calibrated using OxCal v4.4.2 (https://14C.arch.ox.ac.uk/oxcal/OxCal.html)^21^.

A charcoal sample (H15) obtained from faulted unit-1a in the hanging wall yielded a calibrated 2-sigma age of > 43,500 BP is considered to be reworked since unit-1a is faulted and folded, this sample could be derived from adjacent higher surfaces. The unit-3 is the capping unit which post-dates the earthquake event. However, in unit-3, only one sample H25 gave an age range of 347-2 BCE, which does not follow the stratigraphy concerning an underlying unit-1b, and hence it is considered as reworked, which can be derived from the adjacent higher surfaces. Radiocarbon samples obtained from the overbank paleosol unit-1b can be used to bracket the maximum age of the most recent event in the trench. Nineteen radiocarbon samples were dated in the paleosol unit-1b. The samples H3, H30, H32, H34, H35, H37, and H38 are considered to be reworked because of their comparatively older and scattered ages relative to the other samples with respect to its depth within the unit-1b.

If we follow the distribution of the ages, most of the samples from unit 1b show high confidence level (> 60%) of radiocarbon ages spanning between 1445 and 1795 CE (H5, H9, H7, H4, H27, H31, H29) (Table 1, Fig. 5 and Supplementary Fig. S6). However, some samples show a younger set of ages between 1905 and 1943 CE (Table 1, Fig. 5 and Supplementary Fig. S6). It is to note that these younger ages show only 3% of the total 2 sigma ranges, which make these ages least reliable (Table 1, Fig. 5 and Supplementary Fig. S6). The ^14^C results obtained by acid base-acid pre-treatment method, which does not always remove all contaminating carbon compared to wet oxidation method^22^. The measured radiocarbon samples were undergone acid base-acid method, and therefore, a smaller young age ranges may be related to the sample pre-treatment chemistry that can significantly alter the reliability of radiocarbon ages^23,24^.

Primarily, ^14^C ages (or any radiogenic age from detrital components) do not record the age of sediment, but they limit it such that the youngest age provides the maximum age of the sediment^25^. Steier and Rom^26^ have cautioned the use of Bayesian statistics in ^14^C dating, and have suggested to combine with additional scientific information for inferring the timing of an event. Based on these studies^22–26^ a simple interpretation of unit 1b in the trench exposures might conclude that the sediment was deposited after the 1942 CE youngest possible age. The surface-breaking thrust must therefore have followed after the youngest date (i.e. 1942 CE). However, in the present study, we made contrasting approaches of maximum depositional age and a Bayesian approach that seeks to ‘smooth’ the irregularities in what may be a wide range of ^14^C ages.

Albeit large scattering in radiocarbon ages exists, we cannot provide an exact date range for the deposition of unit-1b. However, the face value of the confidence index percentile ages of unit 1b has been used to represent that the earthquake happened after the lower bound calendar age i.e., 1445 CE. Similar interpretations have widely been used in Himalaya by several researchers^12,13,27,28^. Based on the highest probability distribution of radiocarbon ages of samples combined with additional seismotectonic information discussed later in the discussion, we limit the timing of displacement any time after 1445 CE.

Driftwood logs of more than 1 m are observed within a 3–4 m column of sediments and were emplaced in the T2 and T1 terraces (Fig. 2 and Supplementary Fig. S7). We collected two drifted wood log samples (PSB1 and PSB2) from the left bank of Dulung Nala, and one sample PSB3 from the right bank of Chauldhoa Nala (Fig. 2). The geographic locations of these wood logs are shown in Fig. 2 and illustrated in vertical lithologs (Supplementary Fig. S7). The above observations suggest that these wood logs were emplaced in the terrace as a result of massive floods. We dated all three driftwood logs by AMS radiocarbon dating. Sample PSB1 yielded a date range of 1657 to ≥ 1905 CE, and samples PSB2 and PSB3 gave an identical age range of 1674–1943 CE (Supplementary Fig. S7 and Table 1). These date ranges represent the period during which the tree trunks were beheaded and cut-off from the atmosphere. Similar radiocarbon ages for all of the driftwood logs indicate that they were uprooted contemporaneously, i.e., post-1657 CE. We have no control over the fact that these wood logs were emplaced due to an earthquake-induced landslide or the results of a climatic mega-flood event. However, previously published literature indicates that the Subansiri River was dammed for four days following the 1950 Tibet-Assam earthquake^29^ and that the dam subsequently breached, causing massive flooding in the adjoining low-lying areas around the river. Hence, we anticipate that the area was devastated after 1657 CE causing massive landslides and flooding due to a strong earthquake, which resulted in the form of emplacement of these giant tree trunks in the terrace sediments.

The distribution of the majority of the ages obtained from unit-1b places the timing of the initial surface rupture and the creation of the fault scarp after 1445 CE, along the fault strand 'F1′. The fault dips at an angle that varies from 9° to 11° in the trench, which leads to a minimum observed dip-slip displacement of ~ 13 m in the trench^30^ (Fig. 4), but near-surface geophysical imaging indicates that uncertainties in dip beneath the surface must be considered^31^. To incorporate these uncertainties, we assume a dip of 20° to 40° for the causative fault at depth, in which case there is 15.2 ± 4.6 m slip and 13.4 ± 5.3 m shortening is needed to produce the observed vertical separation of 6.8 m. The deduced 15 m slip at our trench site could be an unusual, locally high co-seismic slip, and may also be affected by non-tectonic ground motions. However, we have not seen such indicators in the field that could have helped confirming this hypothesis or quantifying these non-tectonic effects.

## Discussion

Our interpretation of the timing of the most recent earthquake event occurred in the site is relied upon the following additional informations: (i) stratigraphically coherent radiocarbon ages of unit-1b observed based on a simple two-dimensional plot between depth of samples against calibrated radiocarbon ages, and (ii) the maximum number of samples having the highest probability of 2-sigma ages. Following these approaches combining with additional seismological information discussed below, we end up with an earthquake event that occurred after the 1445 CE and most likely in either 1697 CE or 1714 CE.

Alternatively, if we follow the young age model or maximum depositional age, then the earthquake occurred after 1942 CE, and thus point toward 1950 CE Assam Earthquake. However, the 1950 CE event is the first-ever instrumentally recorded earthquake in Himalaya, and its geophysical parameters are fairly established^18,32,33^ baring its surface rupture and co-seismic sub-surface slip. The observed vertical and co-seismic slip at the trenched site along the eastern Himalayan frontal thrust is 6.8 m and 15 m, respectively. The trench exposures do not show multiple colluvium; hence it indicates a single event. The study site is located ~ 200 km west of the 1950 epicenter. Further, previously at the Pasighat trench site (~ 120 km east of present site), Priyanka et al.^17^ have shown a small 3.1 m high fault scarp with a minimum co-seismic slip of 5-m at the eastern Himalayan frontal thrust. Therefore, a large 6.8 m high scarp with 15 m co-seismic slip observed at Himebasti (this study) is unlikely to be the 1950 event as the scarp height decrease toward the rupture termination^12,13,28,34–36^. Finally, the strain calculated from the geodetic convergence rate is known to be more than the moment released by earthquakes, along the Himalayan arc, therefore multiple ~ Mw 8 events within 250 years on the same segment remain rare.

### Segmentation in the eastern Himalaya

Along-strike propagation of surface ruptures in large to great earthquakes along the Himalayan arc is a current topic of discussion. Based on structural considerations, gravity anomaly variations along the arc, and seismicity, it can be argued that major ridges and transverse faults cross-cutting the Himalayan arc govern the along-strike extent of rupture propagation^37–39^. Lateral variability may exist at smaller wavelengths in the form of lateral ramps, for example, and it is possible that while large earthquakes may obey these boundaries, but great earthquakes surpass these discontinuities, rupturing multiple adjacent segments of the fault^40^.

**Figure 6:**
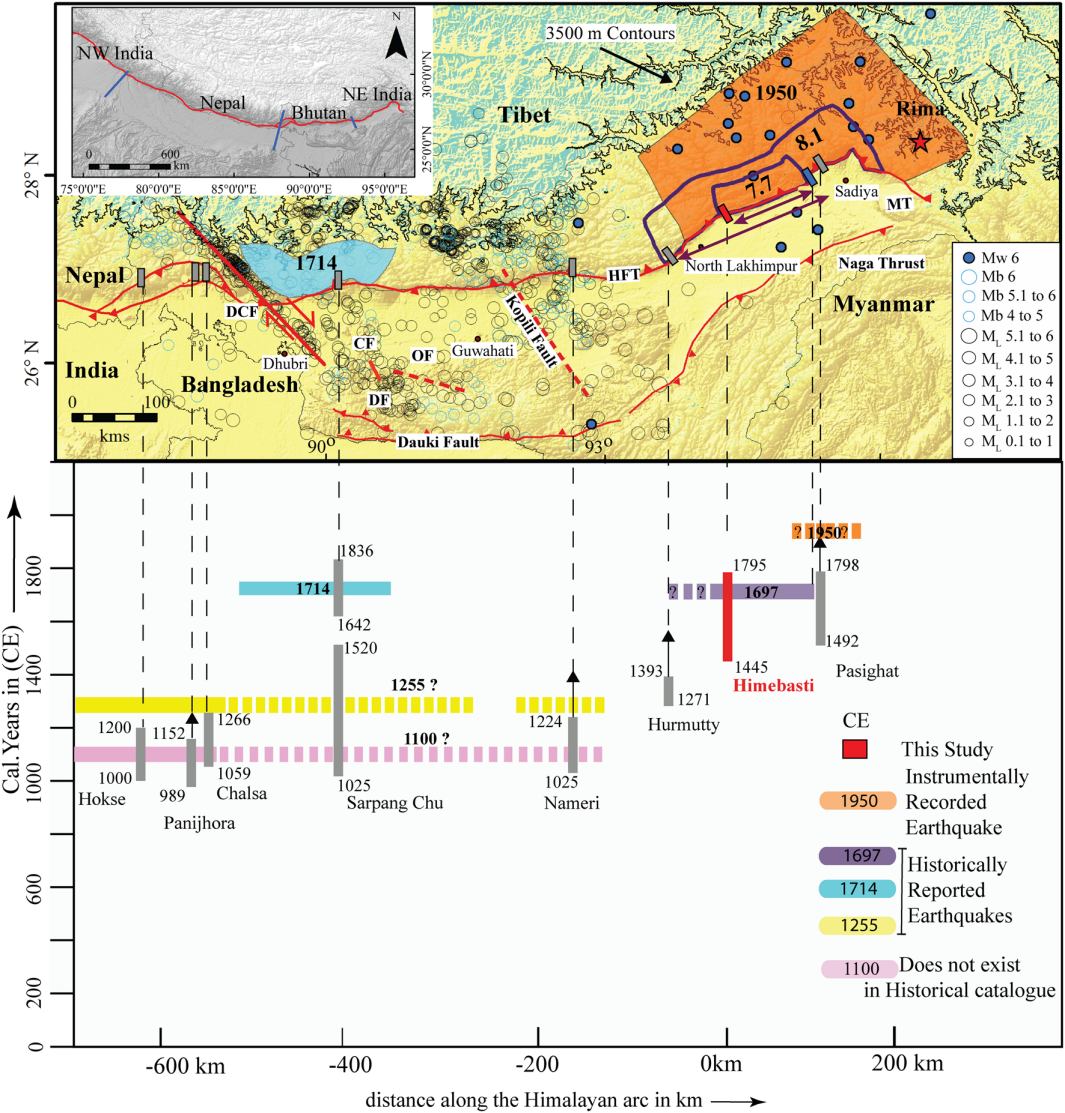
Seismotectonic map and chronology of earthquakes in the eastern Himalaya. Seismicity of the GANSSER project catalogue^39^ and the International Seismological Centre (ISC) catalogue. Blue and orange polygon shows the possible zone for the 1714 event hypocenters from Hetényi et al.^43^ and the proposed rupture zone for the 1950 earthquakes^51^. Blue circles are the 1950 aftershocks having Mw > 6^32^. The blue rectangle is the location of the Nigluk trench^47^ (Priyanka 2018). Trenches (grey rectangles) from west to east are (**a**) Hokse^53^, (**b**) Panijhora^16^, (**c**) Chalsa^13^, (**d**) Sarpang Chu^15^, (**e**) Nameri^13^, (**f**) Hurmutty^13^, (**g**) Niglok^47^, (**h**) Pasighat^17^. A solid red rectangle shows the Himebasti trench. *HFT *Himalayan Frontal Thrust, *DCF *Dhubri Chungthang Fault, *DF *Dapsi Fault, *CF* Chedrang Fault, *MT *Mishmi Thrust. (Inset) SRTM map of the Himalayan arc showing four major transverse structures adapted from Hetényi et al.^38^ (Bottom) Space–time diagram is showing modelled constraints on the timing of occurrence of surface-rupturing earthquakes for a sequence of great medieval earthquakes. The vertical axis is time in calendar years CE, and the horizontal axis is the distance in kilometres from our study area. Maps were prepared in Arc GIS v10.3 using SRTM GTOPO 30 m imagery available at http://glcfapp.glcf.umd.edu/data/srtm/description.shtml. Artwork was done in Adobe Illustrator CS5.

In the eastern Himalaya, Vernant et al.^41^ suggested that a segment boundary exists in the Brahmaputra valley along the Kopili Fault with a 2–3 mm/year dextral slip motion^42^ (Fig. 6). Further, using applied structural concepts, Hetényi et al.^38^ employed arc parallel gravity (APaGA) and topography anomalies (APaTA) to study the along-strike lateral variations of the Himalayan arc and concluded that the Himalaya is divided into four major segments based on flexural geometry. From east to west, they are NE India, Bhutan, Nepal, and NW India through Dehradun (Fig. 6 inset). They further suggested that the Himalayan megathrust earthquakes cannot propagate across these segments and that the easternmost boundary, between Bhutan and the eastern Himalaya, is also focusing seismicity. The next boundary to the west is sharply constrained by Diehl et al.^39^ by a high-quality earthquake catalogue and 3-D crustal P-wave velocity model. Their results locate the fault which separates the Shillong block from the Indian Plate, the Dhubri-Chungthang fault zone (Fig. 6).

**Figure 7:**
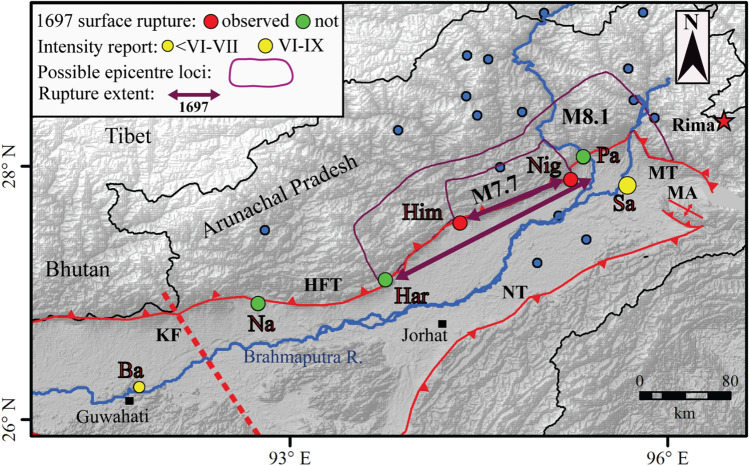
Observations and model results related to the 1697 CE. Sadiya earthquake of a magnitude of 7.9 ± 0.2. Surface rupture information from paleoseismological studies: Him: Himebasti (this study), Nig: Niglok^47^, sites with no observed seventeenth-century surface rupture, according to Kumar et al.^13^ and Priyanka et al.^17^. *Na* Nameri, *Har* Harmutty, and *Pa* Pasighat. Intensity observations are described in the text: *Sa* Sadiya, *Ba* Barnadi Bridge. Possible loci of hypocenters as constrained here are shown for the M7.7 and M8.1 scenarios (purple contours; see text for details). Red star and blue circle, respectively, indicate the location of the 1950 Tibet-Assam earthquake and its aftershocks of Mw > 6, *HFT* Himalayan Frontal Thrust, *MT* Mishmi thrust, *NT* Naga Thrust and *MA* Manabhum Anticline. Map was prepared in Arc GIS v10.3 using SRTM GTOPO 30 m imagery available at http://glcfapp.glcf.umd.edu/data/srtm/description.shtml.

Previous paleoseismological work suggests great earthquakes like the 1100 CE and 1255 CE ruptures may have crossed the Dhubri Chungthang Fault and Kopili Fault^13,16^. However, there is still no consensus on these great earthquakes (Fig. 6). At the Nameri and Harmutty sites, Kumar et al.^13^ correlated the surface rupture event with the 1100 CE event. Considering the above segmentation scenario, earthquakes such as the 1100 CE and 1255 CE events, which have been reported in Kathmandu^9^ and central Nepal, could not reach these study sites. In contrast, a recent seismotectonic study in the Bhutan foothills documented a medieval earthquake followed by an eighteenth-century event^15^. The MRE in their study was correlated with the 1714 CE earthquake and was assigned a 200–300 km rupture length (Figs. 6, 7). Furthermore, using empirical scaling relationships and historical records, Hetényi et al.^43^ estimated a magnitude of 8.0 ± 0.5 for the 1714 CE event. Thus, the extension of medieval earthquakes (e.g. 1100 and 1255 CE) to our trench site is most unlikely. Himebasti village lies adjacent to the Subansiri River, which interestingly lies very close to the high damage zone of the 1697 earthquake estimated by historians^1,44,45^. The locations of the 1714 CE and 1697 CE events are > 400 km apart and are separated by the Kopili Fault, as well as two trench sites (Harmutty and Nameri) where no trace of surface rupture has been documented for the 1714 CE event^13^ (Fig. 6). The 1697 CE and the 1714 CE earthquakes likely happened independently from each other, as suggested by Coulomb stress modelling in the region by Grujic et al.^46^. Hence, we conclude that the MRE in our trench exposure is not the 1714 CE Bhutan earthquake.

### The seventeenth-century event

As referred to by Iyengar et al.^1^, a script named Tungkhungiya Buranji^44^ reports (Supplementary Fig. S8):

"In the month of Puh 1618 (1697 CE), Bandar Phukon of the Chetiya family constructed a fort at Puingdang under the orders of the king, which took two months. In the same year, there was an earthquake, which continued for 6 months in an abortive fashion, from Phagun to Saon (February to August, local names for months in Hindi). The earth was rent asunder at Sadiya, and Magur and Kawai fish appeared in the breaches. As sands and water appeared at the place, the sides of the hills crumbled down".

Reports of massive damage and ground fissuring were reported in nearby Sadiya (Fig. 1), and Iyengar et al.^1^ assigned the event an intensity of at least X on an intensity scale of XII. Our study suggests that the most recent event (MRE) in this segment of eastern Himalaya occurred after 1445 CE. The results of the present study closely correspond to the historically documented 1697 CE Sadiya earthquake that occurred in this eastern Himalayan segment. Rupture imprints of the same earthquake are found at Niglok village along the eastern Himalayan front (95.23° E, 27.89° N)^47^, 97 km east of this site (Fig. 6). The event shook the major cities of the eastern Himalaya and destroyed the town of Sadiya.

### Magnitude estimate of the 1697 CE earthquake

Assuming that the MRE at Himebasti records the imprints of the 1697 Sadiya earthquake, we constrain its possible range of magnitudes based on the available data. The data at hand are shown in Fig. 7 and are composed of: (i) two surface ruptures observed and described at Himebasti and Nigluk, and (ii) two damage reports converted into intensity estimates at Sadiya^44^ and Barnadi^45^. We also include three additional trenches where no surface rupture was observed associated with an event in 1697 CE (Nameri, Harmutty, Pasighat).

Our first estimate is for the minimum magnitude required to produce a surface rupture that extends across the 97 km between Himebasti and Nigluk. We use the scaling relations for thrust faults presented by Wells and Coppersmith^48^:

$$ {\text{M}} = {5}.00 \, + {1}.{22} \times {\text{log}}_{{{1}0}} \left( {{\text{SRL}}} \right), $$

where SRL is surface rupture length in kilometres, and M is magnitude. This constrains the minimum magnitude of the 1697 CE event to Mw 7.4.

In a second estimate, the minimum and maximum magnitude scenarios and possible locations of associated hypocenters are investigated, using all data and methodology presented in Hetényi et al.^43^. This approach includes a map grid search over the range of possible magnitudes. For each point on the map, a binary (0/1) map is created depending on this point fitting (i) rupture length to the two trenches following Wells and Coppersmith^48^ relations between magnitude and subsurface rupture length; (ii) rupture width from the Himalayan Frontal Thrust following scaling relations from the same source; and (iii) observed intensities at the two historically documented sites through intensity prediction equations by Allen et al.^49^, which provides a solution constrained by more parameters than surface rupture length alone. Based on our reading of the historical reports and uncertainties, we assign intensities between VI and IX to the Sadiya site and intensities not exceeding VI–VII at Barnadi (Fig. 7). This approach yields five binary maps for each magnitude and location scenario. When the sum of these five maps displays, the possible hypocenter areas fitting all the observations are directly highlighted (Figs. 6, 7, and Supplementary Fig. S9). This calculation reveals that the minimum and maximum scenarios are Mw 7.7 and Mw 8.1, respectively (Fig. 7). The contours of the corresponding zones are reported in Fig. 7. The likely hypocenter area of the minimum scenario spans between Himebasti village and Nigluk town.

In contrast, the suitable hypocenter area of the maximum scenario spans from Harmutty to the north-easternmost end of the HFT. Magnitude scenarios above Mw 8.1 cannot be excluded, but seem unlikely because of the vicinity of Harmutty and Pasighat sites where there is no observable surface rupture. However, the surface rupture extent of the Mw 8.1 earthquake would still fit between these two sites, as calculated in the equation above. This magnitude range estimate remains valid when other Intensity Prediction Equations are used (another by Allen et al.^49^, as well as one by Szeliga et al.^50^), but the shape of the possible hypocenter locations changes to some extent. The estimates from the two different approaches are congruent and point to the fact that the 1697 CE earthquake most likely had a magnitude of Mw 7.9 ± 0.2 and occurred on the north-easternmost segment of the HFT. Hence, after discussing a wide range of congruent evidence, we conclude that, despite the problems with much scattered and younger 14C ages in the trench, the rupture at Himebasti site could have formed at 1697 CE. The 1697 CE rupture did not surpass the Kopili Fault and therefore occurred on a segment that is different from the section that hosted the 1714 CE earthquake^43^. As such, the 1697 event fits the Himalayan segmentation model proposed in Hetényi et al.^38^. Towards the east, the surface rupture most likely propagated past Niglok site^47^, but not reaching the Pasighat site^17^. Between these two sites, there is a transverse segment that shows an offset of the HFT at around 95.2° E longitude (Figs. 1, 6, and 7), as mapped by Coudurier-Curveur et al.^18^. We propose that the 1697 event has broken the segment reaching the Nigluk site, but it has not jumped to the segment at Pasighat. Therefore, the eastern termination of the 1697 CE rupture is structurally controlled.

## Conclusions

The obtained results demonstrate that this segment of the eastern Himalaya was most likely ruptured by the 1697 CE great earthquake, producing a dip-slip displacement of 15.3 ± 4.6 m. By applying intensity prediction equations and earthquake size scaling laws to the available data, a magnitude estimate between Mw 7.7–8.1 has been calculated for the 1697 CE Sadiya earthquake (Figs. 6, 7, and Supplementary Fig. S9).

The historically documented 1697 CE and 1714 CE earthquakes have unzipped separate segments of the HFT on either side of the Kopili fault, and despite the close time interval of 17 years, they appear to have occurred independently. This means that a given segment of the Eastern Himalaya, of considerable length on the order of ca. 100 km, had ruptured alone in 1697 during an M7.9 ± 0.2 event. Consequently, seismic hazard assessment in the NE Himalaya is very complicated than in a zone with a single, typically linear, major fault, and 3D physical modelling is more keenly needed to model stress and strain evolution. This study adds an important site to the seismic hazard assessment of the eastern Himalaya, which will benefit the inhabitants and help in providing better infrastructure across the region.

## Methods

### Mapping of the Fault scarp

We identified the potential fault scarps and the abundant truncated geomorphic surfaces using Cartosat-1A satellite imageries purchased from http://www.nrsc.gov.in (Source: NRSC, ISRO/DOS). The scarps were mapped using SOCET-GXP software. The mapped terraces and fault scarps were validated in the field by performing an aerial survey using Phantom-2 cod copter or Unmanned Aerial Vehicle (Supplementary Fig. S1). The micro topographic map of the scarp site was generated by the application of high-end survey instruments Real-Time Kinematic Global Positioning System (RTK-GPS) and Robotic Total Station (Fig. 3). The data generated from the RTK-GPS and Total station was further refined using Leica Geo Office (LGO) software.

### 14C dating

Twenty-one charcoal samples were dated from the faulted and un-faulted units to bracket the timing of faulting event in the trench (Fig. 4). All the detrital charcoal fragments were collected in the field from different units. One needs to be careful while sampling because of the modern burned roots of plants and smaller manganese fragments. To avoid any anthropogenic contaminations, we used gloves while collecting the samples. The collected samples were kept in an aluminium foil, and later on, individual charcoals were packed in the zip lock bags to avoid any kind of physical damage to them. The detrital charcoal fragments were analyzed at the Beta Analytic Inc., Miami, Florida. These dates were calibrated using Ox-Cal online programme (OxCal v4.4.2 (https://14C.arch.ox.ac.uk/oxcal/OxCal.html)^21^ with the probability density of 95%. The ages are shown in Table 1 and Fig. 4b.

### Intensity prediction equations (IPE)

This approach includes a map grid search over the range of possible magnitudes. For each point on the map, a binary (0/1) map is created depending on this point fitting (i) rupture length to the two trenches following Wells and Coppersmith^48^ relations between magnitude and subsurface rupture length; (ii) rupture width from the Himalayan Frontal Thrust following scaling relations from the same source; and (iii) observed intensities at the two historically documented sites through intensity prediction equations (IPE) by Allen et al.^49^. Based on our reading of the historical reports and uncertainties, we assign intensities between VI and IX to the Sadiya site and intensities not exceeding VI–VII at Barnadi (Fig. 7). This approach yields five binary maps for each magnitude and location scenario. When the sum of these maps displays five, the possible hypocenter areas fitting all the observations are directly highlighted (Figs. 6, 7, and Supplementary Fig. S9). This calculation reveals that the minimum and maximum scenarios are Mw 7.7 and Mw 8.1, respectively (Fig. 7). The contours of the corresponding zones are reported in Fig. 7. The likely hypocenter area of the minimum scenario spans between Himebasti and Nigluk.

In contrast, the suitable hypocenter area of the maximum scenario spans from Harmutty to the north-easternmost end of the HFT. Magnitude scenarios above Mw 8.1 cannot be excluded, but seem unlikely because of the vicinity of Harmutty and Pasighat sites where there is no observable surface rupture. However, the surface rupture extent of the Mw 8.1 earthquake would still fit between these two sites, as calculated in the equation above. This magnitude range estimate remains valid when other IPEs are used (another by Allen et al.^49^, as well as one by Szeliga et al.^50^), but the shape of the possible hypocenter locations changes to some extent.

## Supplementary Information

Supplementary Information.
