# Supplementary material for: Establishing primary surface rupture evidence and magnitude of the 1697 CE Sadiya earthquake at the Eastern Himalayan Frontal thrust, India
Source: Sci Rep. 2021 Jan 13;11:879. doi: 10.1038/s41598-020-79571-w (PMC7806906; doi:10.1038/s41598-020-79571-w)
Supplement: Supplementary file 1 — Supplementary Information. [file 41598_2020_79571_MOESM1_ESM.pdf]

Supplementary Information

Establishing first surface rupture evidence and magnitude of the 1697 CE Sadiya earthquake at the Eastern Himalayan Frontal thrust, India

Arjun Pandey[1], R. Jayangondaperumal[1, 2*], György Hetényi[3], Rao Singh Priyanka[2], Ishwar Singh[1], Pradeep Srivastava[1], H.B. Srivastava[4]

[1]*Wadia Institute of Himalayan Geology, Dehradun, India*

[2] *Department of Geology, School of Earth Sciences, Central University of Tamil Nadu, Thiruvarur, 610005, India*

[3]*Institute of Earth Sciences, University of Lausanne, Lausanne, Switzerland*

[4]*Department of Geology, Banaras Hindu University, Varanasi, India*

This PDF file includes:

Supplementary figure S1 to S9.

Supplementary figures

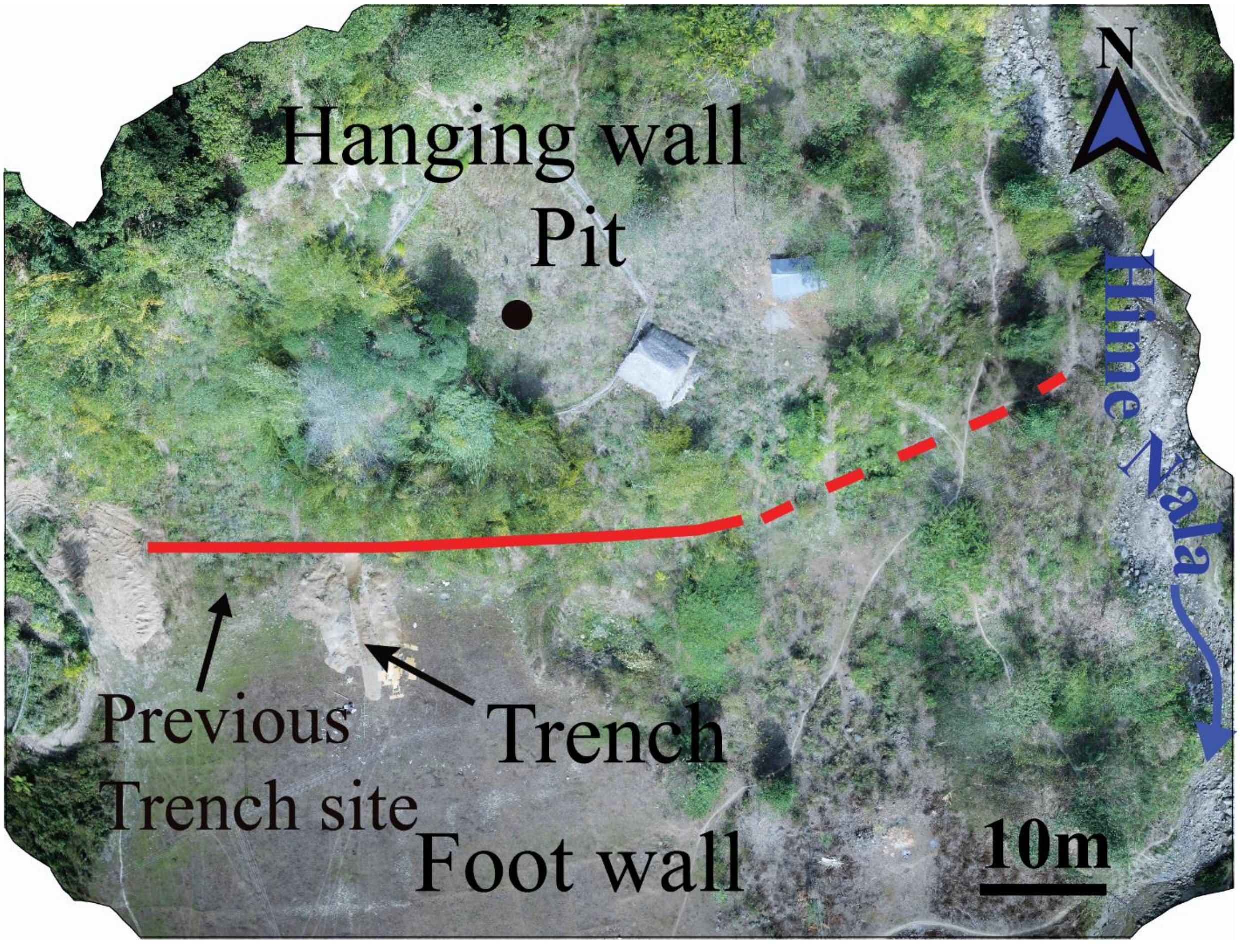

Supplementary figure S1. Aerial view of the Himebasti trench site obtained using Go-Pro digital camera mounted at the bottom of UAV (DJI Phantom 1 multi-rotor cod copter). Redline denotes trace of an active fault. Artwork was done in Adobe Illustrator CS5 software.

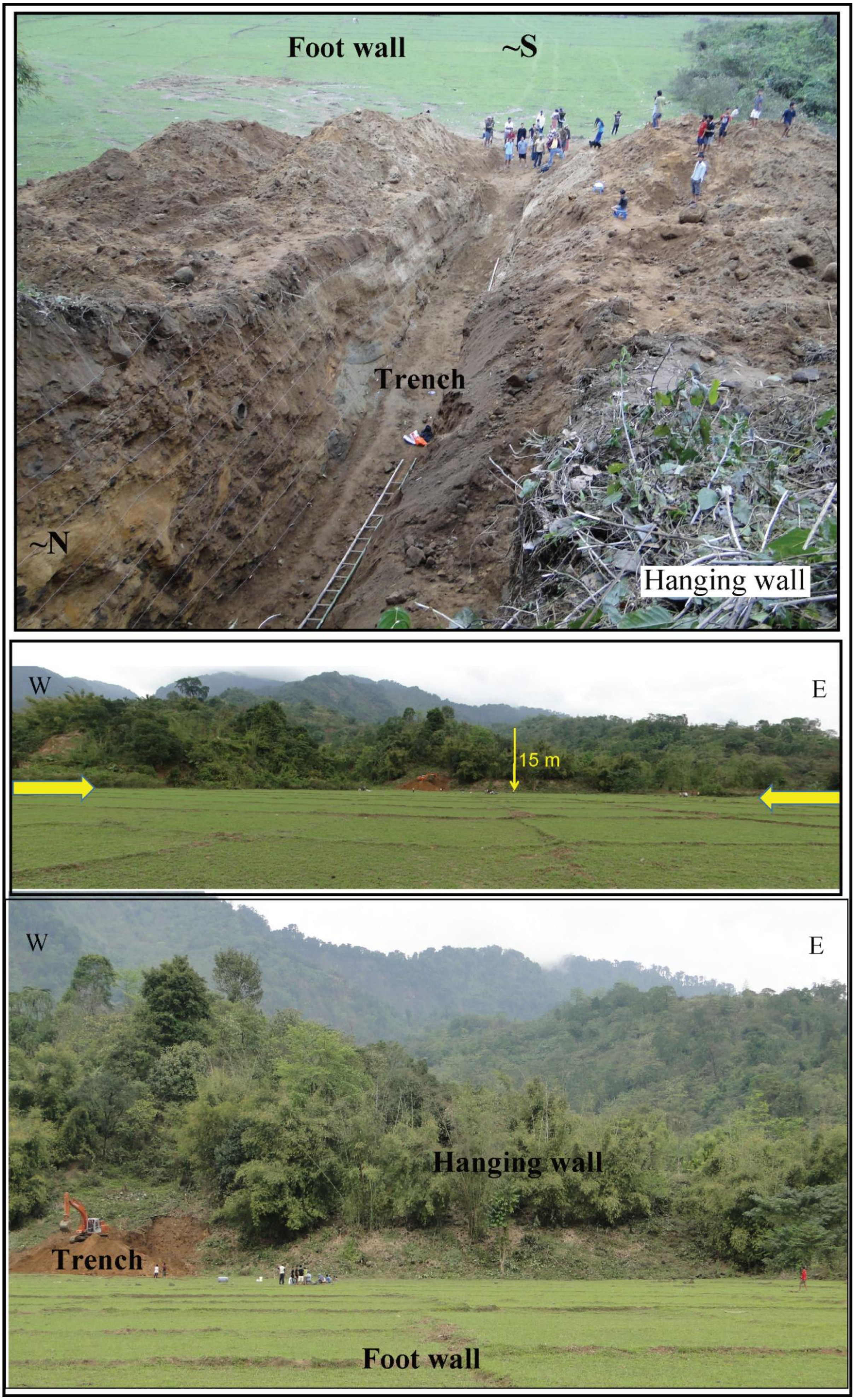
Foot wall
~S
Trench
~N
Hanging wall
W
E
15 m
W
E
Hanging wall
Trench
Foot wall

Supplementary figure S2. (top) Photograph of the excavated trench at Himebasti taken from the hanging wall of the scarp. Field photographs (middle: far, and bottom: close up views) of the trench site showing fault scarp; yellow arrows indicate base of the scarp. Artwork was done in Adobe Illustrator CS5 software.

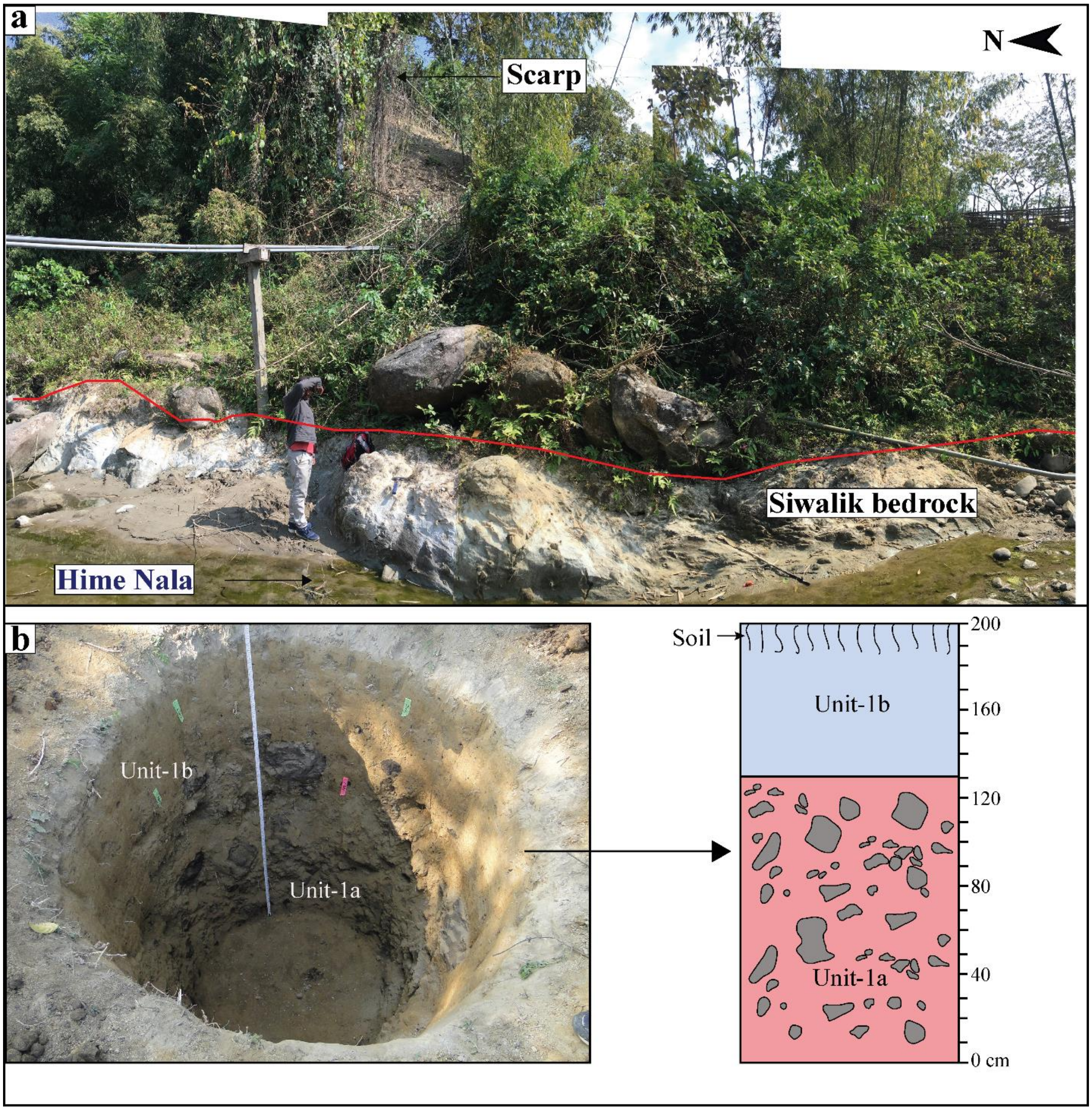

Supplementary figure S3. (3a) Field photographs of the Hime Nala eastern bank (location has been shown in Fig. 2) showing incised Siwalik bedrock (red line-strath contact), suggests the uplift in the area. (3b) Photograph of a pit dug on the hanging wall of the Himebasti scarp. Pit contains two units 1a and 1b, which are matching with the respective units of trench exposures (Fig. 4). The bottom right-hand side is the illustrative log of the excavated pit, and its location is shown in Figs.2, 3 and S1. Figure S3b was prepared in Adobe Illustrator CS5 software.

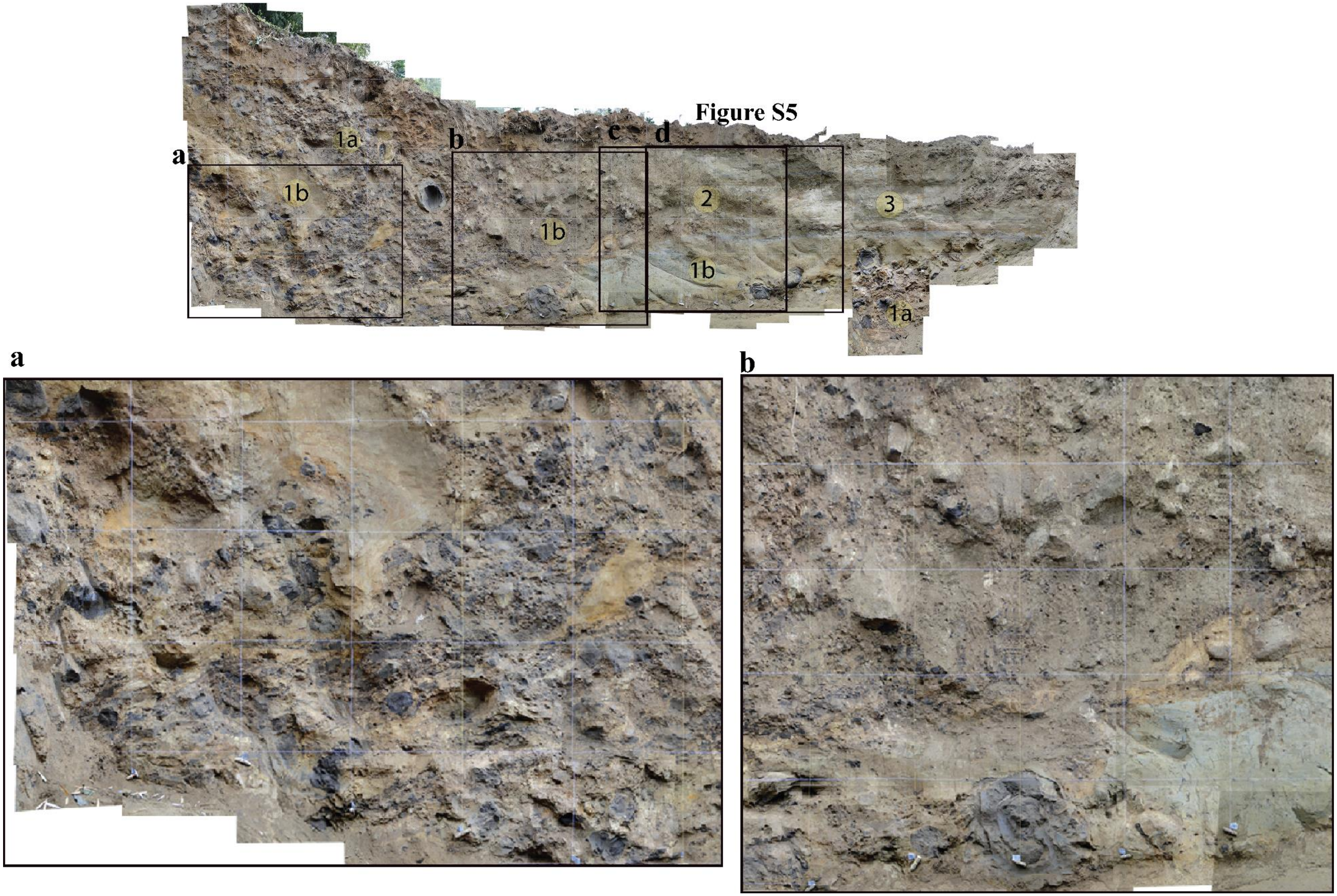

Supplementary figure S4. Photomosaic log of Himebasti trench exposures. Black square box with alphabets ‘a” and “b” are the close

up image shown in the bottom panel that shows an asymmetrical fold in the hanging wall verging toward the south and relation of units 1a and 1b along the fault. Unit numbers are labeled in the yellow circle with numerals. Artwork was done in Adobe Illustrator CS5 software.

c

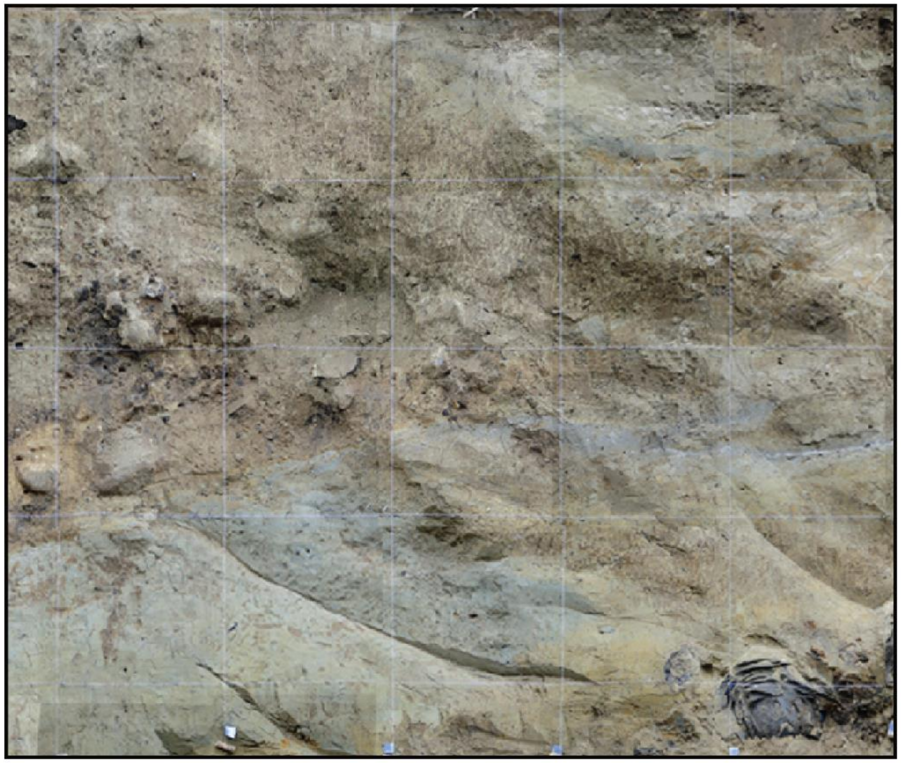

d

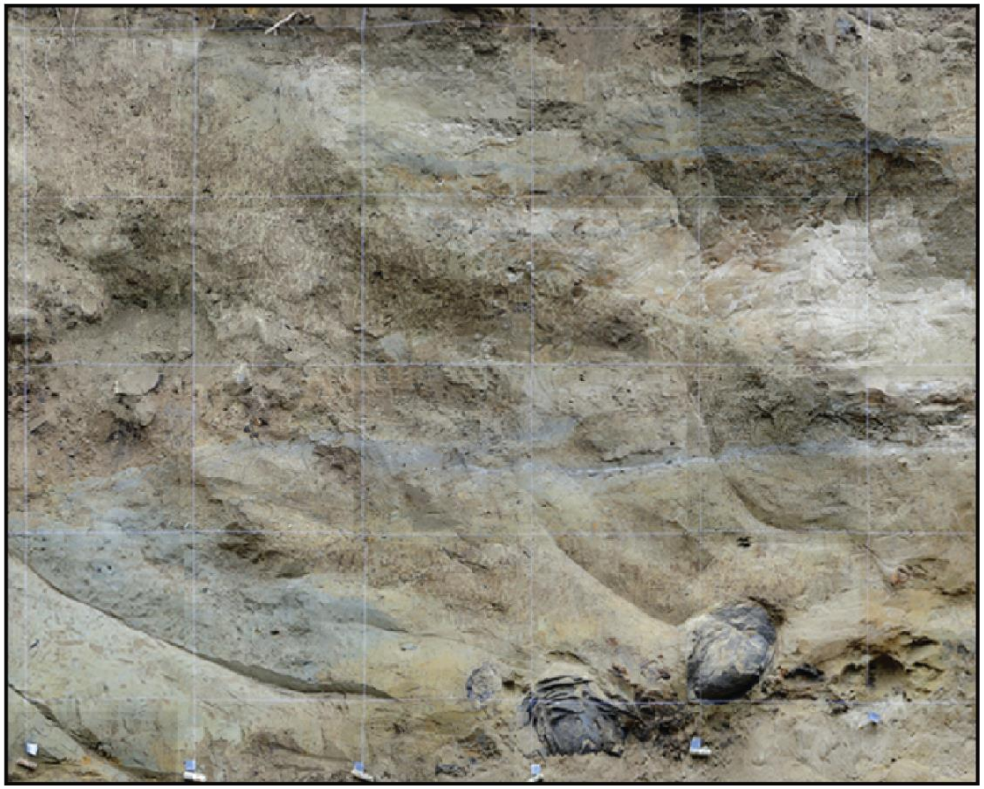

Supplementary figure S5. Magnified photograph of the footwall of the Himebasti trench. The location of these photos are shown in Sup. Fig-4 (top panel). The enlarged photos show the bulldozing of unit-1a and 1b and the colluvium unit 2 with fault contact. For unit reference and location, refer to Supplementary Fig.S4.

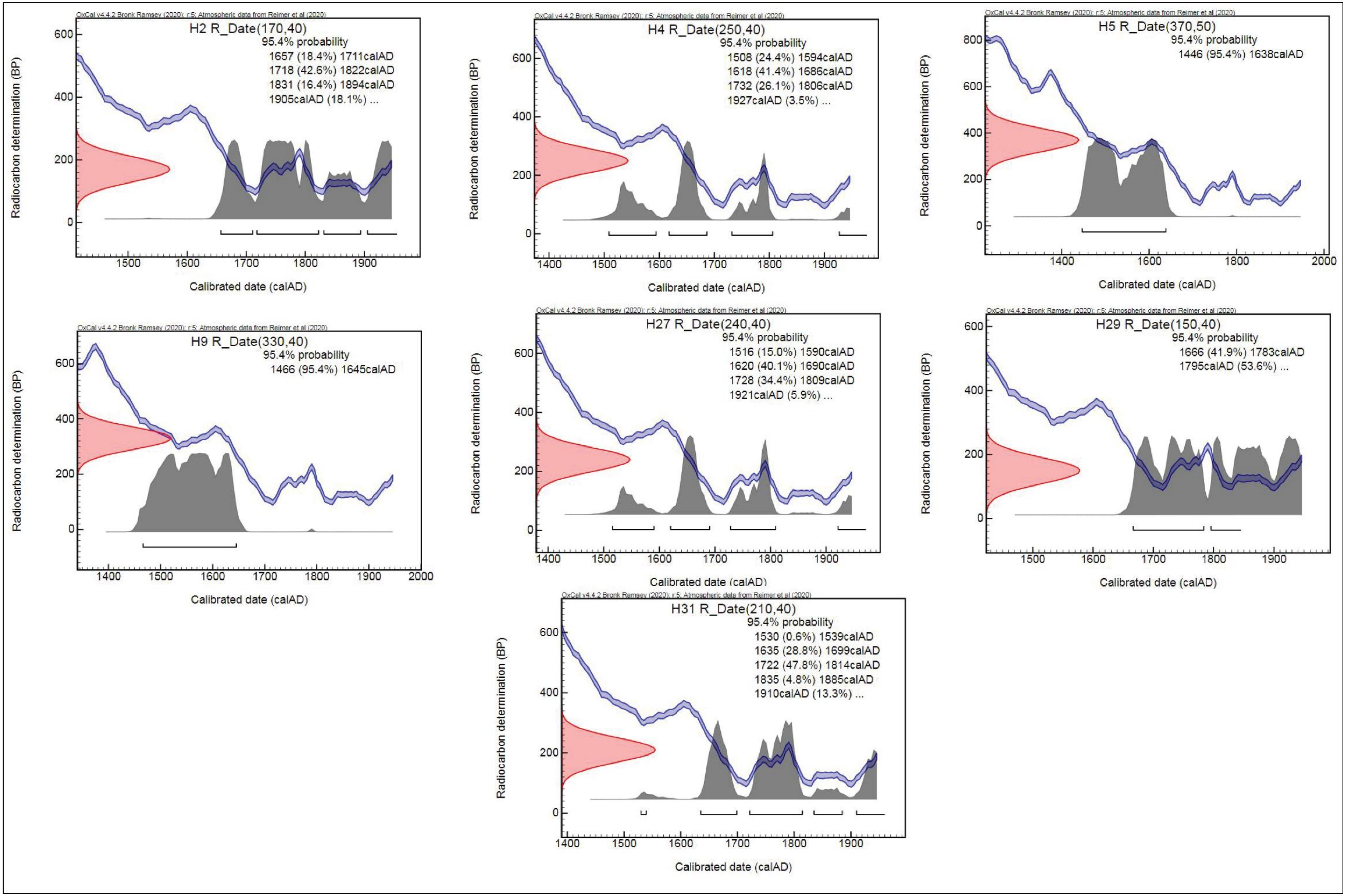
OxCal v4.4.2 Bronk Ramsey (2020); r:5; Atmospheric data from Reimer et al (2020)
H2 R_Date(170,40)
95.4% probability
1657 (18.4%) 1711calAD
1718 (42.6%) 1822calAD
1831 (16.4%) 1894calAD
1905calAD (18.1%) ...
Radiocarbon determination (BP)
Calibrated date (calAD)
OxCal v4.4.2 Bronk Ramsey (2020); r:5; Atmospheric data from Reimer et al (2020)
H4 R_Date(250,40)
95.4% probability
1508 (24.4%) 1594calAD
1618 (41.4%) 1686calAD
1732 (26.1%) 1806calAD
1927calAD (3.5%) ...
Radiocarbon determination (BP)
Calibrated date (calAD)
OxCal v4.4.2 Bronk Ramsey (2020); r:5; Atmospheric data from Reimer et al (2020)
H5 R_Date(370,50)
95.4% probability
1446 (95.4%) 1638calAD
Radiocarbon determination (BP)
Calibrated date (calAD)
OxCal v4.4.2 Bronk Ramsey (2020); r:5; Atmospheric data from Reimer et al (2020)
H9 R_Date(330,40)
95.4% probability
1466 (95.4%) 1645calAD
Radiocarbon determination (BP)
Calibrated date (calAD)
OxCal v4.4.2 Bronk Ramsey (2020); r:5; Atmospheric data from Reimer et al (2020)
H27 R_Date(240,40)
95.4% probability
1516 (15.0%) 1590calAD
1620 (40.1%) 1690calAD
1728 (34.4%) 1809calAD
1921calAD (5.9%) ...
Radiocarbon determination (BP)
Calibrated date (calAD)
OxCal v4.4.2 Bronk Ramsey (2020); r:5; Atmospheric data from Reimer et al (2020)
H29 R_Date(150,40)
95.4% probability
1666 (41.9%) 1783calAD
1795calAD (53.6%) ...
Radiocarbon determination (BP)
Calibrated date (calAD)
OxCal v4.4.2 Bronk Ramsey (2020); r:5; Atmospheric data from Reimer et al (2020)
H31 R_Date(210,40)
95.4% probability
1530 (0.6%) 1539calAD
1635 (28.8%) 1699calAD
1722 (47.8%) 1814calAD
1835 (4.8%) 1885calAD
1910calAD (13.3%) ...
Radiocarbon determination (BP)
Calibrated date (calAD)

Supplementary figure S6. Individual probability density plot of radiocarbon ages for the charcoal samples procured from the Himebasti trench. These samples show least probability percentile for the 19th and 20th century age span (e.g. H2, H4, H27 and H31), while 95.4% probability percentile ages ranging between 1445 – 1645 CE. The plot has been generated in the OxCal v4.4.2 (https: / /c14 .arch .ox .ac .uk /oxcal /OxCal .html[23]) with the IntCal20 atmospheric curve of Reimer et al., 2013[34]. Artwork was done in Adobe Illustrator CS5 software.

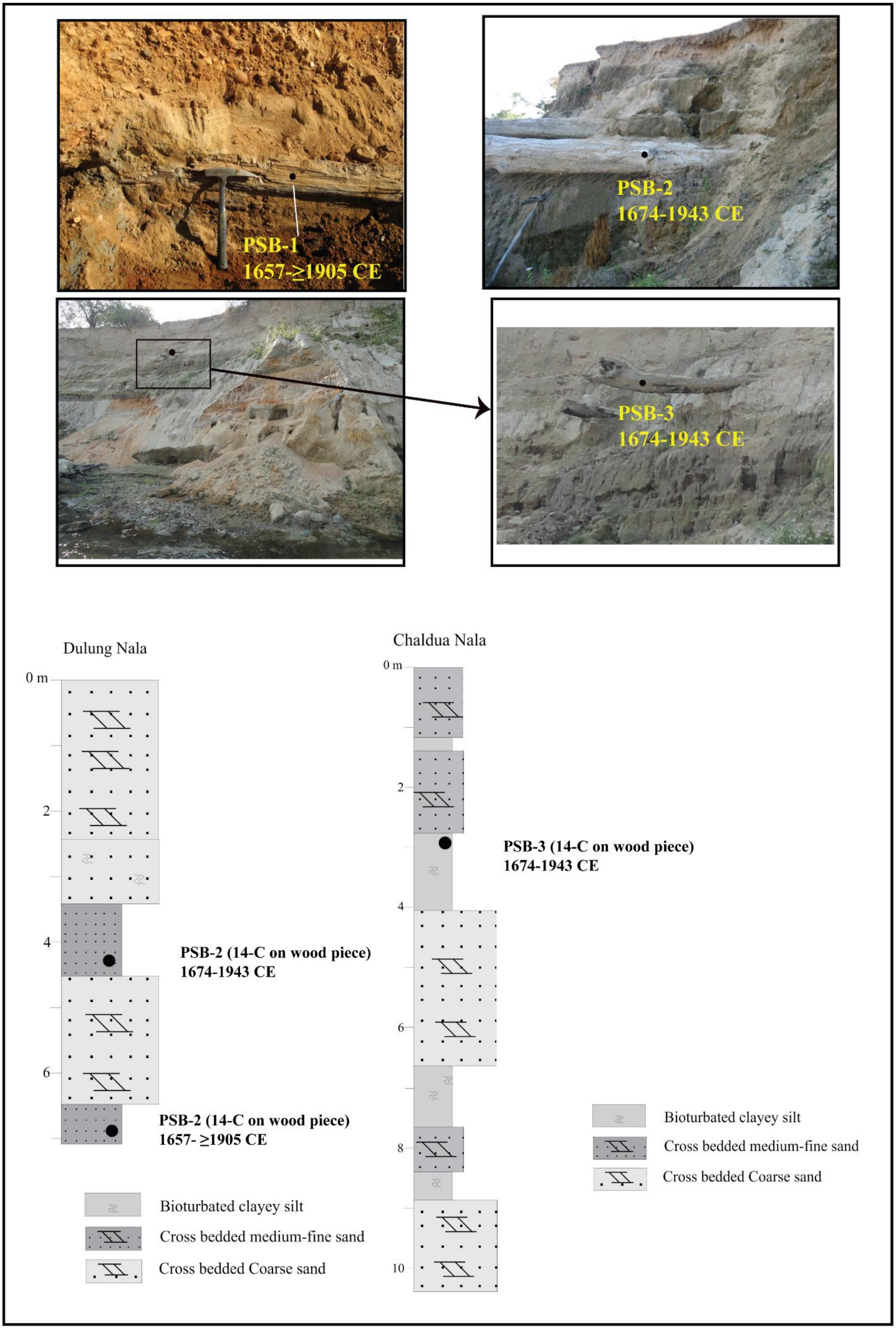
PSB-1
1657-≥1905 CE
PSB-2
1674-1943 CE
PSB-3
1674-1943 CE
Dulung Nala
0 m
2
4
6
PSB-2 (14-C on wood piece)
1674-1943 CE
PSB-2 (14-C on wood piece)
1657- ≥1905 CE
Bioturbated clayey silt
Cross bedded medium-fine sand
Cross bedded Coarse sand
Chaldua Nala
0 m
2
4
6
8
10
PSB-3 (14-C on wood piece)
1674-1943 CE
Bioturbated clayey silt
Cross bedded medium-fine sand
Cross bedded Coarse sand

Supplementary figure S7. (Top**)** Field photographs of the wood log samples PSB-1, PSB-2 and PSB-3 emplaced in the terraces. The locations of these samples are shown in Fig 2 of the main manuscript. The respective lithologs of the samples are illustrated in the lower panel. Bottom: Lithologs of the river cut sections from where the wood log samples PSB-1, PSB-2, and PSB-3 were procured. Lithologs have been prepared in Adobe Illustrator CS5 version.

Earthquake.

In the month of Puh, 1618, Bandar Phukan of the Chetia family constructed a fort at Pungdang under the orders of the King which took two months. In the same year there was an earthquake which continued for six months in an abortive fashion, from Phagun to Saon of the following year. The earth was rent asunder at Sadiya, and *magur* and

30 TUNGKHUNGIA BURANJI.

kawai fish appeared in the breaches. As sands and waters appeared at that place the sides of the hills crumbled down. 53.

Supplementary figure S8: In the historical chronicle Tungkhungia Buranji[48] chapter IV, page 29-30 reports. Reports of massive damage and ground fissuring were reported in nearby Sadiya, and Iyengar et al. (1999)[1] assigned the event an intensity of at least X on an intensity scale of XII.

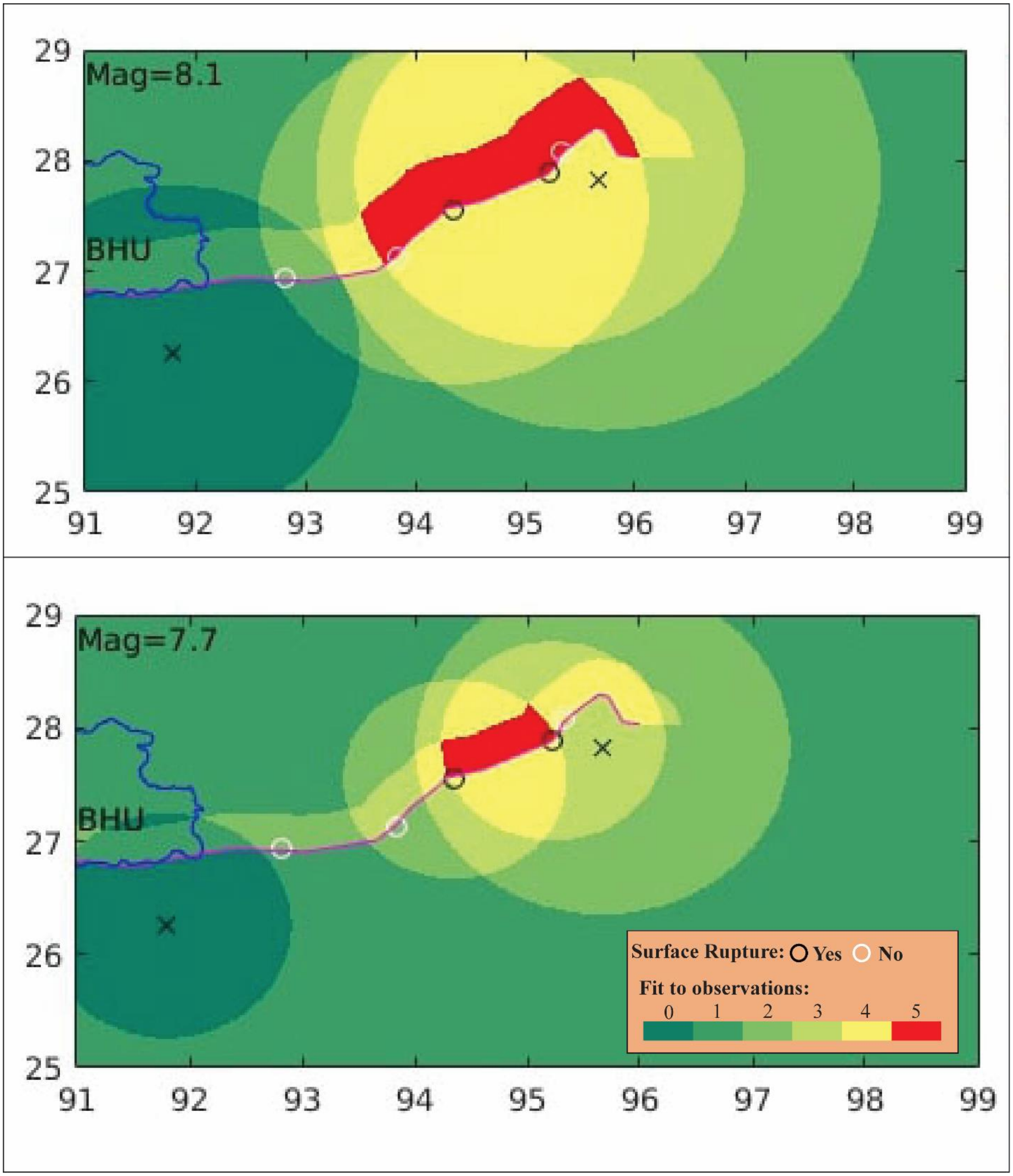

Supplementary figure S9. Combining historical damage and paleoseismological surface rupture observations, to the intensity prediction equation by Allen et al., 2012[52] and scaling relations by Wells and Coppersmith (1994)[51]**.** As explained in the text, (**Top**) shows maximum magnitude, M8.1, and (**Bottom**) shows minimum magnitude, M7.7 scenarios. The red area represents hypocenter locations fitting with observed data. BHU; Bhutan. Maps were prepared using MATLAB software version 2016b. Artwork was done in Adobe Illustrator CS5 software.
